# Learning from the rDNA Operon: A Reanalysis of the *Acanthamoeba palestinensis* Group

**DOI:** 10.3390/microorganisms12102105

**Published:** 2024-10-21

**Authors:** Daniele Corsaro

**Affiliations:** CHLAREAS, 54500 Vandoeuvre-lès-Nancy, France; corsaro@gmx.fr

**Keywords:** *Acanthamoeba* T2/T6, rDNA operon, LSU rDNA, ITS-2, phylogenesis

## Abstract

The molecular classification of *Acanthamoeba* is currently based on the analysis of 18S rDNA sequences, delimiting around twenty genotypes (T1–T23). In some cases, however, the resolution of 18S is limited, and other genetic markers could be useful for unravelling poorly resolved lineages. In this study, the partial large subunit (LSU) of rDNA and ITS were used to re-examine the *Acanthamoeba palestinensis* group (T2/T6 lineage), which consists of various poorly defined lineages, including the T2 and T6 genotypes. New sequences overlapping 18S, ITS, and LSU were recovered. The analysis placed previously identified partial ITS-LSU sequences as T2/T6 and further confirmed the separation of the OX1 lineage from T2. In addition, analysis of the second internal transcribed spacer (ITS-2) suggests that multiple species may be present within the T6 and OX1 lineages. The results obtained from the T2/T6 lineage analysis confirm the utility of partial LSU and ITS for the study of *Acanthamoeba*, suggesting their advantage for disentangling complex lineages.

## 1. Introduction

*Acanthamoeba* spp. (Amoebozoa, Discosea, Centramoebida) are free-living amoebae, widespread in any environment, and are of particular interest because they can behave as opportunistic parasites for humans and animals. Amoebae can enter through open wounds or the respiratory tract and spread to other tissues and organs and ultimately to the brain, causing fatal sub-chronic granulomatous amoebic encephalitis (GAE). Ocular infection mainly affects the cornea, with amoebic keratitis (AK) leading to loss of vision [1].

*Acanthamoeba* classification currently relies on the analysis of the complete nuclear small subunit (SSU) ribosomal RNA (rDNA) gene, recognising more than twenty genotypes/subtypes (T1–T23, T4A–T4H) [2,3,4]. The genotypes thus identified correspond in part to the classical species, classified according to the characters of the double-walled cysts in three morphological groups, MG1 to MG3 [5,6], as well as to possible new species that remain to be described. The traditional morphological classification is less and less used because it is considered unreliable. This is true at the species level and particularly for those of MG2. In many cases, however, genotypes and morphological groups correspond. There is convincing evidence that many strains have been wrongly assigned to different species of *Acanthamoeba*, which requires the reconsideration of morphological characters in light of genetic data [3,7]. Complete nuclear SSU rDNA (18S rDNA) allows for the outline of a general phylogeny of *Acanthamoeba*. However, its resolving power is limited, and disentangling finer relationships remains difficult, especially for lineages that, despite low 18S divergence, appear very diverse.

In a wide variety of organisms, improved results have been obtained using other portions of the rDNA operon, such as the internal transcribed spacer (ITS) region, composed of the 5.8S rDNA flanked by ITS-1 and ITS-2, or the large subunit (LSU) of rDNA, particularly its domains I and II [8,9]. These portions have been previously analysed for various *Acanthamoeba* genotypes, with promising preliminary results [10].

One lineage that would be interesting to analyse in more detail is that of *Acanthamoeba palestinensis*. This group consists of two closely related genotypes, T2 and T6 [2], which originally included three MG3 strains: the type strains of *A. palestinensis* (Reich) [11] and *Acanthamoeba pustulosa* (GE3a) [5] in T2, and “*A. palestinensis*” strain 2802 [12], originally identified as *Acanthamoeba lenticulata* [5], in T6. However, it was subsequently shown that various MG2 strains misassigned to other species also cluster with the T2/T6 clade in 18S trees, some of which emerge as intermediate lineages [13]. The *A. palestinesis* group, or T2/T6 lineage, in fact, includes a mixture of diverse species from different morphological groups, often still wrongly labelled. The reference sequences of the intermediate lineages are those of three strains MG2, Page-45 (ATCC 30872/CCAP 1501/3B) from a lake [11] and OX-1 (CCAP 1501/3C) from an old distilled water bottle [14], both isolated in the USA and originally assigned to *Acanthamoeba polyphaga*, and *Acanthamoeba* sp. KA/MSG27, from ocean sediments near South Korea [15]. Other MG2 strains, such as the pathogenic 11DS, causing keratitis and wrongly attributed to *Acanthamoeba hatchetti* [16] or the environmental CDC-149, instead belong to T6. The story of strain CDC-149 is a typical example of misidentification. This amoeba was isolated as a host of *Legionella* in a hospital cooling tower and microscopically identified as *Comandonia operculata* [17]. Subsequent molecular analyses revealed an 18S rDNA of *Acanthamoeba*, also noting a close resemblance in fine structure to *Acanthamoeba* strain Neff, a typical MG2 strain [18]. Instead of raising the issue of possible misidentification, the early morphological diagnosis was assumed to be reliable, leading some to synonymise *Comandonia* with *Acanthamoeba* and to the completely unjustified proposal of *Acanthamoeba operculata*. In contrast, *Comandonia* should be considered a junior synonym of *Flamella* (Amoebozoa, Variosea) given the nearly identical morphology/structure of *C. operculata* as originally described by Pernin and Pussard [19] with strains of this amoeba, including the WBT strain originally identified as *C. operculata* [20].

In a previous study [10], using the complete rDNA operon recovered from the *A. palestinensis* genome, still erroneously registered in GenBank as that of *Acanthamoeba healyi*, it was possible to identify partial ITS-LSU sequences deposited in GenBank as most likely belonging to one or the other lineage of the T2/T6 group. Their clear assignment was, however, hampered by the lack of 18S. In the present study, additional sequences were recovered that overlap 18S, ITS and LSU, thus filling some gaps and allowing a more complete re-examination of the T2/T6 lineage.

## 2. Materials and Methods

The complete rDNA operon of *A. palestinensis* strain Reich (CDFA01), as well as other species, has already been obtained from genomes available on the NCBI/GenBank portal [10]. It should be noted that in this portal, many genomes are still wrongly named (last accessed on 24 September 2024), although their true strains/species have been identified [3]. The 18S sequences of T2/T6 strains were retrieved from GenBank based on literature data as well as BLAST searches. The ITS-LSU part includes ten partial sequences deposited in GenBank as uncultivated fungi but previously shown to be *Acanthamoeba* T2/T6 [10]. Additional sequences were identified as putative members of T2/T6 for their 18S match in long-read metadata from a soil metagenomic study. The sequences obtained are partial rDNA operons, including the ITS region and the partial 18S and LSU sequences (Ac_5737: SRA:ERR2355432.5737.1; Ac_7776: SRA:ERR2355433.7776.1; Ac_8539: SRA:ERR2355433.8539.1; Ac_9385: SRA:ERR2355433.9385.1).

In order to reliably confirm the sequence identification and to draw a basic tree topology, first, an analysis was performed on the 18S rDNA. The consistency of the relationships was then analysed for the other parts of the rDNA operon. Sequences of T4 species were used as outgroups. Multiple alignments were performed using MAFFT v. 7 (L-INS-I option) [21] and manually refined using BIOEDIT 7.7 [22]. Molecular phylogenetic analyses were performed as previously described [13,23]: Maximum Likelihood (ML) (GTR *Γ*+I:4) trees were constructed using TREEFINDER 2011 version [24], and the global topology was checked with Neighbour-Joining (NJ) (Kimura 2-P) and Maximum Parsimony (MP) using MEGA7 [25], with 1000 bootstraps. BIOEDIT was used to obtain pairwise similarity values for rDNA sequences. Mean values within and between groups were calculated manually. Secondary structures of the ITS-2 were built using Mfold [26] at the UNAFold Web server (http://www.unafold.org).

## 3. Results

### 3.1. Sequence Identification by 18S rDNA Phylogeny

Most of the 18S sequences included in the analysis are nearly complete (>2200 bp). However, several of them contain indels and/or modified sites, probably due to sequencing errors. These are indeed incompatible with the 2D reconstructions of the regions concerned because they result either in mismatches between the paired stems (e.g., strain EI5) or in the loss or insertion of parts of one or more of their components (e.g., EFW strains). For the sequences newly recovered from soil metadata, the 18S portion is about 1670 bp (75% of the complete sequence), which includes most of the hypervariable regions allowing their identification.

**Figure 1:**
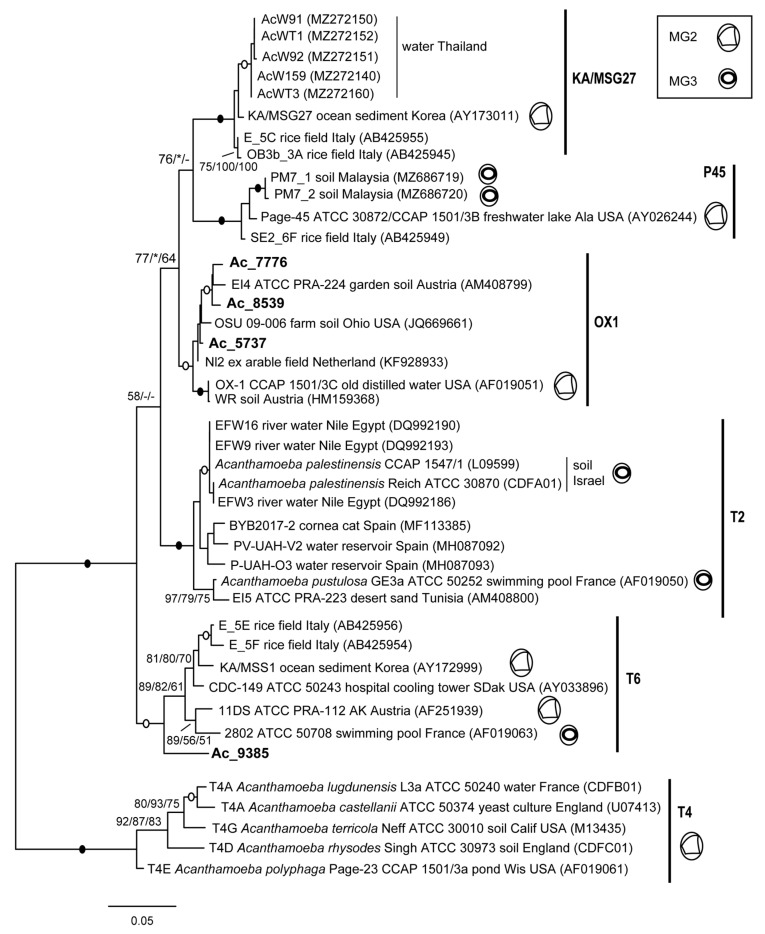
Molecular phylogeny of *Acanthamoeba* T2/T6 group based on complete 18S rDNA. The five groups are indicated and, when available, the cyst type, i.e., MG2 (polymorphic endocyst with arms; ectocyst usually wrinkled) and MG3 (round endocyst; ectocyst usually smooth) [5,6]. Newly recovered sequences are in bold. Members of T4 are used as outgroups. At nodes, bootstrap values (1000 replicates) for ML/NJ/MP, with filled and open circles for values 100 or >95% with all methods. *, node recovered but support <50%; -, node not recovered.

Phylogenetic analysis shows five distinct lineages within T2/T6, generally well supported and differing from each other by approx. 4%, although their precise relationships remain unclear (different results depending on the tree method and/or outgroup used). Two of them correspond to the T2 and T6 genotypes, while the others could be putative new genotypes, as previously suggested [13]. Three partial sequences from the soil metadata, Ac_5737, Ac_7776 and Ac_8539, clearly belong to the OX1 lineage, while a fourth sequence, Ac_9385, can be assigned to the T6 genotype (Figure 1).

The OX1-like sequences show >97.7% similarity with OX-1, while the T6-like sequence is 96.2% similar to strain 2802, and all differ by 4.2–5.3% from *A. palestinensis* (94.7–95.8% similarity).

### 3.2. Partial LSU rDNA Analysis and Phylogeny

**Figure 2:**
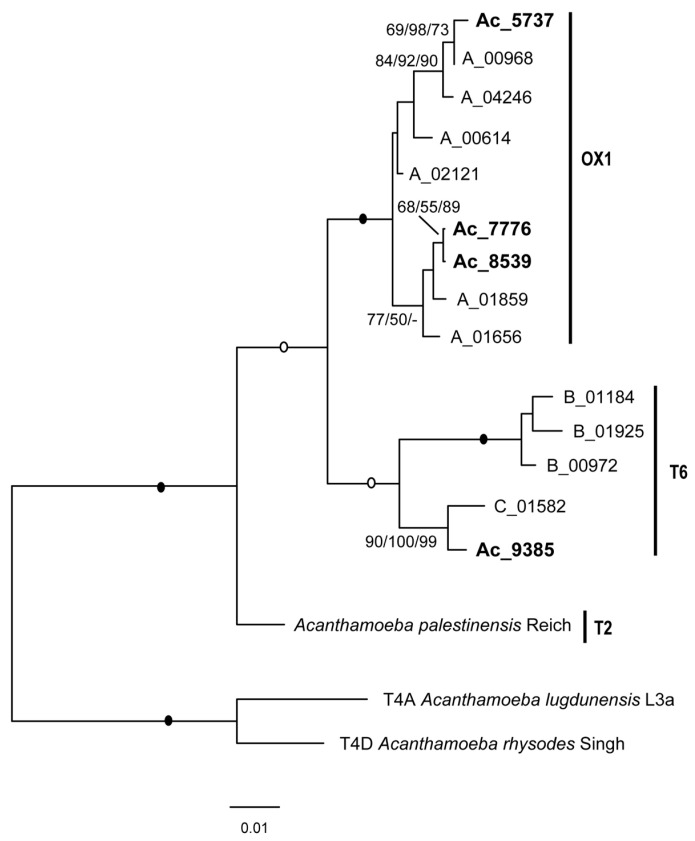
Molecular phylogeny based on partial LSU rDNA. For the T2/T6 group, the sequences analysed are those of T2, T6 and OX1. Newly recovered sequences are in bold. Sequences with prefixes “A_” to “C_” refer to previously identified groups A to C [10]. *Acanthamoeba* T4 is used as an outgroup. Bootstrap values at nodes as in Figure 1.

The LSU portion of the new OX1- and T6-like sequences is approximately 3000 bp long and spans domains I to IV (up to stem 71) (numbering after Petrov et al. [27]). It was therefore possible to confirm the presence of an AU-rich expansion segment between stems 55 and 54 on the 3′ side (domain III) ranging from 62 to 72 nt with an AU content of 61.1 to 68.2% (59 nt for *A. palestinensis* with 69.5% AU [10]). This AU-rich segment should be the hidden break site, allowing the *Acanthamoeba* LSU rRNA to split into two fragments, 26Sα and 26Sβ [10,28,29]. The availability of well-defined OX1- and T6-like LSU sequences also allowed us to confirm and further identify three groups of partial ITS-LSU sequences (shorter LSU portion, <1300 bp, corresponding to domains I/II), labelled A to C, previously identified as related to *A. palestinensis* and assumed to be T6 and one of the intermediate lineages [10]. Indeed, it is now possible to affirm that group A belongs to the OX1 lineage, while groups B and C are both part of T6 (Figure 2).

Partial LSU similarity values between the three OX1-like sequences range from 97.2 to 99.4% and from 98.2 to 99.6% between them and group A. The T6-like sequence, Ac_9385, has a higher similarity (98.2%) to the 01582 sequence (group C) than to group B (<95%), which forms a distinct lineage. The values between OX1 and T6 types are 94.1–94.7% and 94.6–95.6% between these and *A. palestinensis*.

### 3.3. ITS Analysis and Phylogeny

It has been previously shown that in *Acanthamoeba*, ITS-1 and ITS-2 vary considerably in length, mainly due to multiple short repeats [30], but generally, ITS sequences from closely related lineages are of similar sizes [10]. For the T2/T6 lineage, a four-size distribution can be observed based on ITS-1/ITS-2 lengths, distinguishing, respectively, *A. palestinensis* T2, the *Acanthamoeba* sp. OX1 group and two groups within *Acanthamoeba* sp. T6. It is interesting to note that the 5.8S rDNA of the OX1 lineage is 162 nt, as in the other *Acanthamoeba* species, while that of *A. palestinensis* is 160 nt, whereas it is 163 or 164 nt depending on the T6 group. These differences are due to the loss of two nucleotides (T2) or the addition of one or two nucleotides (T6) in the loop of the GC-rich stem B8 (H9 in the Petrov et al. [27] numbering) of the 5.8S secondary structure. In addition, T6 is also characterised by a compensatory base change (C-G to U-A) in the B8 stem, not observed in the other genotypes.

**Figure 3:**
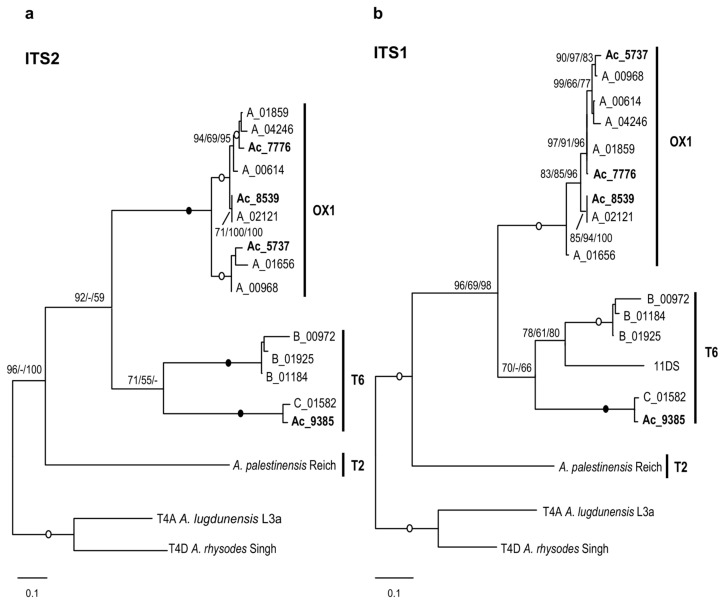
ITS phylogeny. (**a**) Molecular phylogeny based on the 5.8S rDNA/ITS-2. (**b**) Molecular phylogeny based on the ITS-1.

Phylogenetic analysis based on 5.8S rDNA plus ITS-2 fully confirms the results obtained with LSU, showing even better resolution and stronger statistical support for internal nodes with high intragroup similarities (>95%). *A. palestinensis* forms a distinct lineage, while the OX1 lineage appears to be composed of several subgroups (ITS-2 similarity of about 80%), and two distinct groups can be recognised within T6 (Figure 3a). A similar tree topology is also recovered using ITS-1 (Figure 3b). Internal relationships for OX1 are slightly modified, but the closest sequences still show high similarity values (>97%). For T6, similarity values are low, except for Ac_9385 and Ac_01582 (93.4%). Strain 11DS, for which ITS-1 is also available (GenBank ID AF526427) [30], turns out to be the sister of group B (mean similarity 68.7%), whose sequences show only about 86.5% similarity to each other and <60% to the others.

### 3.4. ITS-2 Structure

**Figure 4:**
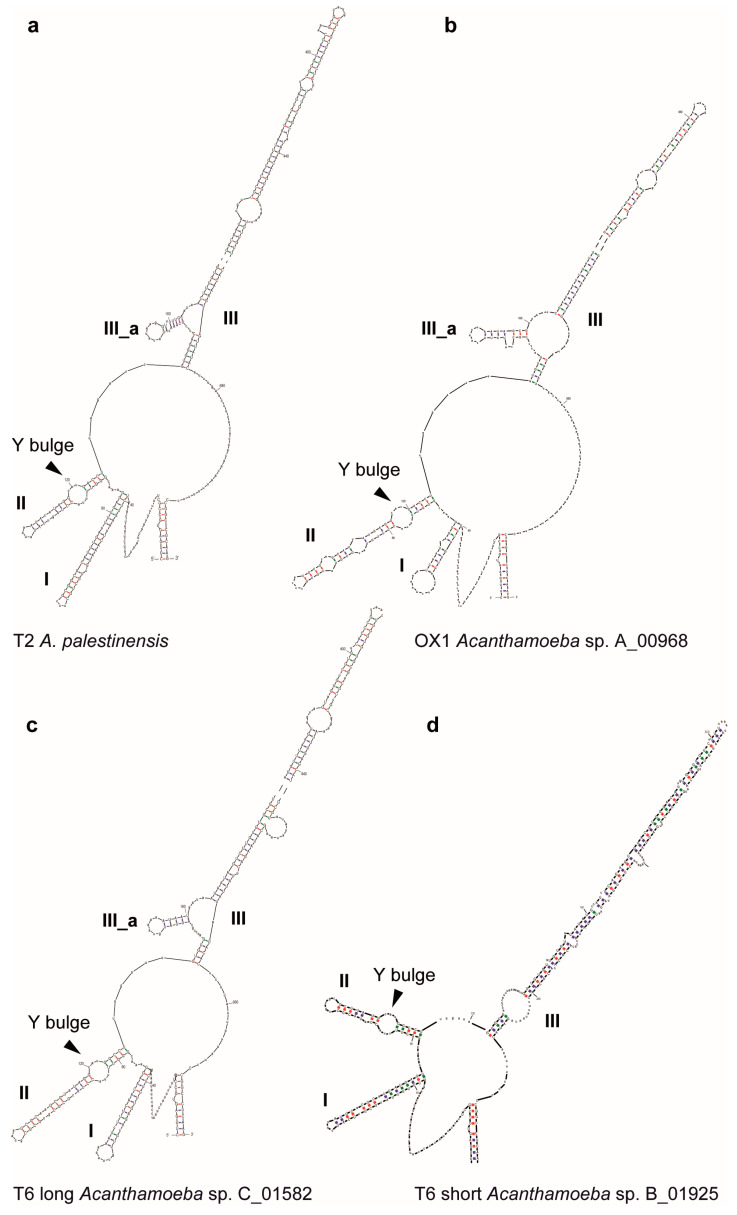
ITS-2 secondary structure. Note that for the T6 short only, the structure is complete. For the others, helix III was cut in the middle. Y bulge: pyrimidine–pyrimidine mismatch.

The overall structure of the ITS-2 of *A. palestinensis*, the OX1 lineage, and both T6 groups is very similar but differs in primary sequence motifs, consistent with the level of divergence observed in phylogenetic trees. All form three-helix structures, from I to III, with helix IV always absent (Figure 4).

Helix II is recognisable by the typical C/U mismatch, producing a bulge near the base of the stem. Helix III is particularly long, about 380–500 nt, and has an additional small helix IIIa at its base of about 20 nt. Within T6, however, group C differs in having a relatively short helix III of only 140 nt and no helix IIIa (Table 1).

**Figure 5:**
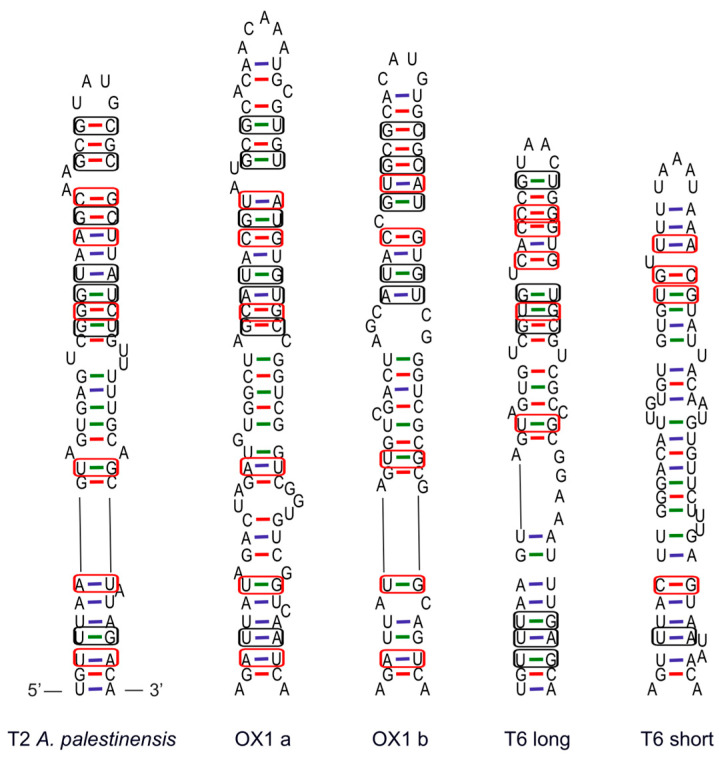
Terminal 30 nucleotides on the 5′ side of helix III of ITS2 from *A. palestinensis* (T2) and the other T2/T6 lineages analysed. The red box indicates compensatory base changes; the black box indicates unilateral (hemi-compensatory) base changes. Note the great divergence between the lineages and the difficulty in identifying homologous sites.

Inspection of the terminal portion of helix III, particularly the last 30 nt on the 5′ side, reveals strong variation between lineages (Figure 5).

## 4. Discussion

**Figure 6:**
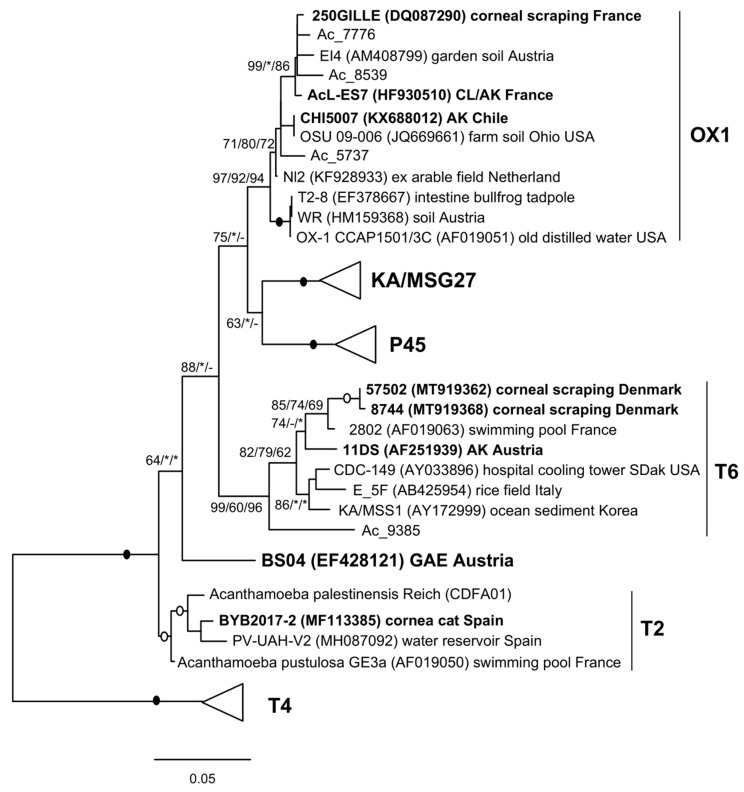
Molecular phylogeny based on partial 18S rDNA. The analysis includes partial 18S fragments spanning the hypervariable region of the gene. Sequences from clinical samples are in bold. Collapsed groups and bootstrap values at nodes are the same as in Figure 1.

The *A. palestinensis* group (clade T2/T6) has a wide geographic distribution but appears to be relatively rare in the environment. This contrasts with the abundance of other genotypes, more frequently found in clinical samples, especially T4. However, cases of AK [16,31,32,33,34,35] and even GAE [36] identified as T2 or T6 have been reported. It has previously been proposed to divide the T2 genotype into two groups, T2A and T2B, because some pathogenic T2 isolates from AK cases in Iran were genetically distinct from the non-pathogenic strain OX-1 CCAP 1501/3C [31]. This situation has become more complex, with the analysis of other strains suggesting that additional groups, distinct from both T2 and T6, can be delineated [13]. Interestingly, the few strains found in AK cases and typed as T6 or T2 can indeed be assigned to either T6 or, more precisely, to the OX1 group. The T2 genotype appears non-pathogenic, with the exception of one case associated with an ocular infection in a cat. It is also noteworthy that the only known case of GAE, typed T2 [36], does not cluster directly with either group and could represent an additional lineage (Figure 6).

Analyses based on 18S (Figure 1 and Figure 6) therefore indicate that T2/T6 is divided into several lineages and show that most pathogenic strains belong to either T6 or OX1. This is further confirmed by the analyses of partial LSU rDNA (Figure 2) as well as ITS-1 and ITS-2 (Figure 3). It is indeed clear that the OX1 group is not part of T2 but forms a distinct lineage, and all data consistently indicate high variability within T6 and OX1. Unfortunately, clearer relationships could not be established due to a lack of data from other groups. Furthermore, for T2, a complete rDNA operon is available for *A. palestinensis* but only the 18S for *A. pustulosa*. The latter has often been considered a synonym of *A. palestinensis* due to isozymes, rDNA restriction profiles, and high 18S similarity [2,12,37]. However, *A. pustulosa* forms a distinct branch in phylogenetic trees (Figure 1), and it would therefore be interesting from a taxonomic point of view to analyse its ITS-LSU.

ITS-2 is used as a barcode and guide to delimit species, usually on the basis of compensatory base changes in homologous parts of the molecule that otherwise have identical sequences. In many organisms, even a few changes are correlated with reproductive incompatibility [38,39,40]. However, the analysed *Acanthamoeba* sequences differ between lineages and exhibit a greater number of variable sites and indels in homologous parts, particularly on the 5′ side of the helix III end (Figure 5), which is expected to be highly conserved. Such variation in ITS-2 could indicate the presence of multiple species, which would be consistent with the 18S and LSU data indicating that each lineage corresponds to a distinct species. In particular, the clear difference between the long and short ITS-2 in T6 strongly supports their separation into two “species”.

The ITS-LSU portion undoubtedly provides useful data for the phylogenetic analysis of *Acanthamoeba* with possible implications in taxonomy and diagnosis. It could be particularly interesting for species-rich lineages, especially those with low 18S genetic distances, such as the T4 subtypes. The analysis of additional sequences from other genotypes will allow for a better appreciation, especially of ITS-2, to distinguish species.

## Figures and Tables

**Table 1:** ITS-2 helix lengths of the *A. palestinensis* group T2/T6.

GT	Species	Group	ITS2 Helix Length (nt)				
			I	II	IIIa	III	IV
T2	*A. palestinensis*	Reich	46	38	23	500	–
OX1	*Acanthamoeba* sp.	(A)	28	54	15–25	370–390	–
T6	*Acanthamoeba* sp.	long (C)	36–46	50	16–17	500–505	–
		short (B)	37–54	36–41	–	140	–

## Data Availability

The original contributions presented in the study are included in the article, further inquiries can be directed to the corresponding author.
